# Conspiracy Beliefs Are Related to the Use of Smartphones behind the Wheel

**DOI:** 10.3390/ijerph18157725

**Published:** 2021-07-21

**Authors:** Pedro M. Valero-Mora, Juan José Zacarés, Mar Sánchez-García, María Teresa Tormo-Lancero, Mireia Faus

**Affiliations:** 1 Departamento de Metodología de las CC del Comportamiento, Universitat de València, Blasco Ibáñez, 21, CP 46010 Valencia, Spain; 2 Departamento de Psicología Evolutiva y de la Educación, Universitat de València, Blasco Ibáñez, 21, CP 46010 Valencia, Spain; juan.j.zacares@uv.es (J.J.Z.); mmsanche@uv.es (M.S.-G.); 3 Independent Researcher, CP 46010 Valencia, Spain; M.Teresa.Tormo@uv.es (M.T.T.-L.); mireia.faus@uv.es (M.F.)

**Keywords:** smartphones, developmental psychology, adulthood, risk behaviour, driving

## Abstract

The belief in conspiracy theories predicts behaviors related to public health such as the willingness to receive vaccines. This study applies a similar approach to an aspect of road safety: the use of smartphones while driving. A representative sample of 1706 subjects answered a series of questions related to what can be regarded as erroneous or conspiracy beliefs against restricting or banning the use of smartphones while driving. The results show that those having such conspiracy beliefs reported a greater use of smartphones behind the wheel.

## 1. Introduction

The field of research into the belief in conspiracy theories as a social psychological phenomenon is rapidly growing [1]. Conspiracy theories can be defined as “explanatory beliefs about a group of actors that collude in secret to reach malevolent goals” [2]. Van Prooijen and Jostmann [3] point out four relevant aspects of these theories: “conspiracy theories are consequential as they have a real impact on people’s health, relationships, and safety; they are universal in that belief in them is widespread across times, cultures, and social settings; they are emotional given that negative emotions and not rational deliberations cause conspiracy beliefs; and they are social as conspiracy beliefs are closely associated with psychological motivations underlying intergroup conflict.”

Often associated with conspiracy theories is a lack of confidence in science or scientific results that has been linked to deficiencies in analytical thinking ability [4], statistical thinking biases [5], or the need for “cognitive closure” [6]. One of the most important consequences that belief in conspiracy theories has is the rejection of public health measures such as vaccination [1].

One problem that has many similarities with the rejection of vaccines is the opposition to the road safety measures promoted by the governments of the countries. Thus, it is common to observe certain degrees of distrust on the part of some sectors of the population both towards the alleged effectiveness of the measures and of the actual reasons underlying the efforts behind their promotion—with the implication that there are ulterior motives. This distrust often materializes in statements that go against the statistical evidence or the recommendations of the experts, such as that seat belts can be dangerous for drivers because “one can fall into a lake and not have time to unfasten it”; that airbags “do more harm than good in a crash”; that helmets are only capable of protecting “something that has the shape of a head in the laboratory but not in real accidents”, so forcing the people to use them is an infringement on their personal liberties [7]; or that speed surveillance cameras are “tax cameras” [8]. For example, Morelock et al. [9] studied the opposition and support for the mandatory seat belt laws in New England and signaled that “despite the many efforts of public officials to alter the public’s knowledge about seatbelt effectiveness, it appears from these analysis that many common myths about seatbelts have not been dispelled”. Examples of what they regarded as myth about seat belts —given the scientific evidence—were: as follows “It is better to be thrown clear of the car if you are in a serious accident”—14% of the respondents strongly thought this was true—or “A person who is wearing a set belt is less likely to be injured in a serious accident than a person who does not wear one”—31% believed this. Their data also showed that the people supporting those myths were more inclined to oppose a mandatory belt use law, used seatbelts less, reported more heavy drinking and smoking marijuana before driving, and had more crashes than the rest of the population [9]. In the past, these kinds of beliefs have constituted the basis for opposing the enactment or the application of legislation supporting certain road safety measures, despite the evidence of the benefits that they would bring in terms of crashes and casualties. The lack of confidence in the road safety measures often affects only small percentages of the population, but, unfortunately, the media usually magnifies the real opposition and fosters the false perception that the measures are unpopular with the public at large [10].

It has been found that exposure to scientific evidence about the road safety benefits of some laws increased the support towards them, but in some subjects this “treatment” had the opposite effect such that they wound up being less supportive or more oppositional after such exposure [11].

Currently, one important issue in road safety is the distraction caused by electronic devices in general and more specifically by the use of smartphones behind the wheel. Although the evidence regarding the negative consequences of their use and their involvement in crashes [12,13,14,15,16,17,18] is overwhelming, with the consequence that restrictions or bans have been imposed in many countries in the world, the surveys continue to show that its use is still widespread [19,20,21,22].

Yeo and Park [23] mentions that the psychological factors that predict the use of the smartphone behind the wheel are normative beliefs of cellphone use, perceived risk of accident, perceived risk of fines, attitudes toward using cellphones while driving, and perceived importance of calling [23,24,25,26,27]. Kaviani et al. [28] explores nomophobia, or smartphone addiction [23], as another factor of importance, particularly for young people [29,30]. Similar to other safety measures, laws banning smartphone use while driving are not as effective as desired [31,32], and they may even have worsened the problem according to Jannusch et al. [30], as their data showed that, in Germany, young novice drivers continue to use it for texting, but in a more dangerous manner, as their effort to conceal this behavior adds an additional load to the visual, cognitive, and physical that it already carries over.

A cause of the lack of effectiveness of the laws banning the use of the smartphones behind the wheel may be that those who engage in it may find justification in conspiracy theories that discredit the claims about the risks implied. This justification would use similar narratives to those that are applied to other road safety measures such as speed cameras [8] or seatbelts [9]. These narratives usually touch the topics of skepticism towards the recommendations themselves, monetary motives [33], infringement of personal liberties [9], false government-expertise based on cooked up evidence [8], or lack of trust in science [11]. People using the smartphone behind the wheel would use claims such as “ it is not as dangerous as they would have us believe”, or “the people who really poses danger are left unthreatened by a police force preoccupied with harassing motorists”, or that fines are “just concealed taxes” so that they could keep a ‘law abiding citizen’s self-righteous image despite their real behavior [8].

The objective of this study is twofold. First, we will undertake an evaluation of the existence of conspiracy theories about the claims regarding the risks of using smartphones while driving, and secondly, we will test the relationship between these beliefs and the reported use of smartphones. The hypothesis that is maintained in this case is that this relationship is positive and that those who most commonly hold this belief use their smartphones behind the wheel more frequently than those who do not.

## 2. Design and Method

This study shows the results of a survey carried out in Spain on the behaviors of smartphone users and the uses the smartphones are put to while driving. The questions in this survey were developed by members of our research team who subsequently consulted with an external team specializing in communication and field studies. The conspiracy theory questions were created trying to reflect opinions or expressions actually used in both the media and internet forums and were agreed upon after several rounds of review. The rest of the questions also went through a similar review process.

### 2.1. Participants and Procedure

The sample used in this study consisted of 1706 subjects, stratified by sex, age, and region in Spain. The profile of the interviewees consisted of subjects with a driver’s license and access to computers. A company specializing in survey research and with extensive experience in the field was in charge of contacting the participants and processing the data.

The surveys were obtained using the CAWI (Computer-Assisted Web Interviewing) methodology. The subjects received an invitation to participate in a study into smartphones behind the wheel and received a link to access the questions.

Table 1 shows statistics describing the sample used in this study. There were 924 women (54%) and 782 men (46%) in the sample. The group age with more participants was the one ranging between 25 and 34 years old (32%), followed but the one between 25 and 34 years old (27%) and the one between 45 and 54 (21%). Fifty-three percent of subjects had university studies, 39% high school studies, and 7.7% primary, and only a small percentage (0.3%) did not have any time of academic title so we merged the last two categories in the subsequent analysis. Ninety-four percent of the participants drove cars and the rest motorbikes (2.9%), vans (1.8%), bus (1.1%), and trucks (0.4%).

### 2.2. Measures

The survey started with questions about demographic variables (age, gender, educational level, etc.), followed by others about habits of smartphone’s use behind the wheel, what specific uses the participants made of the smartphone when driving, and whether they agreed with a series of statements related to what we regard as conspiracy beliefs rejecting the risks of using smartphones while driving. These last two sets of questions will be described in the next subsection.

#### 2.2.1. Statements Regarding Conspiracy Beliefs

Statements regarding conspiracy beliefs are found in Table 1. There are eight statements to which respondents had to indicate agreement or disagreement. Of these statements, four of them (1, 2, 3, and 8) could be considered to denote a rejection of the accepted knowledge that using the smartphone influences the ability to drive; two of them (4 and 6) imply a distrust towards the official sources in charge of promoting road safety in the country; one of them (5) refers to a broader unspecific mistrust not related to road safety, and, finally, one statement (7) could be considered an anomalous or distorted belief about supposed reasons why using the smartphone when driving is not dangerous.

Cronbach’s α for this scale was 0.69, which is close to the recommended value of 0.7. Dropping individual items of the scale did not increase the total value of α.

#### 2.2.2. Questions about Smartphone Use

Table 2 lists the questions about the frequency of use of the smartphone behind the wheel throughout the 12 months up to the time the participants responded to the survey. These questions used a scale of 1 to 5 where 1 = Never, 2 = Almost never, 3 = Sometimes, 4 = Almost always and 5 = Always, and enquired whether the subject had performed any of the indicated tasks.

Using a device inside a vehicle can produce three fundamental types of interference with driving: (1) cognitive, (2) visual, or (3) manual. Depending on the activity, the interference may be mainly of one type or incorporate several types in varying proportions. For example, answering a call using the hands-free phone device (question d) has fundamentally cognitive elements. On the other hand, answering using the phone’s speaker (question c) has a slight manual component in addition to the cognitive, and looking at who is calling without taking the call (question a) produces mainly visual interference. The manual element is greater if the call is answered without the hands-free device (question b). Other questions have manual, visual, and cognitive interference components: for example, using the smartphone’s internet browser (question h) or taking selfies or making recordings with the mobile (question i) all imply the three types of interference with the driving task.

Cronbach’s α for this scale was 0.83, which is over the recommend value of 0.7.

### 2.3. Data Analysis

The responses to the two group of questions were analyzed separately. Frequencies and percentages of positive responses broken by gender, academic level and age were calculated for the positive responses to questions in Table 2, with chi-square or Fisher’s exact tests of significance calculated in each case and Holm’s adjusted post hoc pairwise proportion tests displayed only if the former tests were significant. Means and standard deviations of the responses measured by the same variables as above were calculated for the responses to the questions in Table 3, with *t*-tests or analysis of variance tests depending on the number of categories of the independent variables and Games–Howell post hoc tests using the Tukey’s correction shown if they were significant.

A structural equation model with two latent variables, one of each drawn from the group of questions in Table 2 and Table 3, plus gender, age, and level of studies as manifest variables, was calculated in order to examine the hypothesis that conspiracy beliefs about smartphones might predict their use behind the wheel. The model was calculated using lavaan [34], with use of the smartphone set as an ordinal variable.

## 3. Results

The results include a summary of the responses to the questions on conspiracy ideas about the risks of using smartphones at the wheel and about their use by the respondents. These two aspects will be analyzed as a function of demographic variables (gender, academic level, and age) as well as with a regression analysis with latent variables to test whether conspiracy beliefs predict the use of the smartphone behind the wheel.

### 3.1. Conspiracy Beliefs about the Risks of Using the Smartphone behind the Wheel

Table 4 shows the results for the questions on conspiracy beliefs regarding mobile phone use behind the wheel listed in Table 2.

The first column of Table 4 shows the percentage of people in the sample who agreed with each of the statements. The minimum and maximum values are 3.9% for question 8 (If you go alone on the road and it is straight there is no danger in using your smartphone) and 11.5% for question 5 (You must not turn on your smartphone when you drive because, if you do so, they can pinpoint your location). Notice that although these percentages do not appear too high for individual questions, the percentage of subjects who agree with at least one of these statements rises until it reaches 30% of the sample.

Breaking down the percentages by gender, we observe that men present significantly higher percentages of agreement than women in the case of question 4 (Fines for using the smartphone are only for tax collecting purposes) (10% versus 4.9%, *p* <0.0001) and for question 6 (The statistics of accidents caused by smartphone use are all made up) (6.1% vs. 3.4%, *p* = 0.009). Finally, the percentage of men who agreed with at least one of the statements was also significantly higher than that of women (33% vs. 27%, *p* = 0.02).

The academic level of the subjects was associated with differences in responses to question 2 (I do not think that looking at the smartphone makes you drive worse) (*p* <0.001). The subjects with a secondary or primary academic level expressed greater agreement with this statement than those with a tertiary (university) level. The same thing happened for questions 3 (You do not lose sight of the road when using your smartphone behind the wheel) (*p* = 0.013), 5 (You do not lose sight of the road when using your smartphone behind the wheel), (*p* = 0.003), 6 (By not being able to use the smartphone, people become more bored when driving which leads to more accidents) (*p* = 0.009), and 7 (By not being able to use the smartphone, people become more bored when driving which leads to more accidents) (*p* < 0.001). Finally, the varying academic levels also led to significant differences regarding the percentage of subjects who agreed with at least one of the statements. This was the case for 41% of the subjects with a primary education level and for 33% of high school students versus 26% of those that reached a tertiary level of education.

Finally, age was a variable that did not show differences in terms of conspiracy beliefs except in the case of question 2 (I do not think that looking at the smartphone makes you drive worse), in which 14% of the subjects in the age group 55 or over indicated agreement versus only 5.8% of those aged 25–34 and 5.4% of those aged 35–44.

### 3.2. Reported Use of the Smartphone behind the Wheel

The first column of Table 5 shows the means of the responses for all the subjects in the sample. The behavior that obtained the highest value was d (Answer or make a call using the car’s hands-free device) ($\bar{x}=2.5$), followed by a (Seeing who is calling you, without actually taking the call) ($\bar{x}=2.1$). In the first case, although many people regard this behaviour as lo-risk, the research shows that it can be cognitively challenging, and experts recommend that it be avoided as much as possible. The type of behavior to which the second question refers involves mainly visual distraction, but also manual if the smartphone has to be reached from within a bag, compartment, etc.

Regarding gender, significant differences are observed in item c (Answering or making a call by putting on the smartphone speaker) in which women have higher means and in d (Answer or make a call using the car’s hands-free device), in which it was the men who had higher values.

The differing academic level led to differences in item e (Reading a WhatsApp message or something similar or an email), in which university-level students showed a higher mean than those of the primary level, but there were no differences with the other group, and differences in item i (Taking selfies or making recordings with the smartphone), in which those with secondary education scored higher averages than those of university level.

The participants reported different frequencies of use of the smartphone behind the wheel in 9 out of the 10 statements in Table 2 as a function of their age (with the exception being g (Interacting (checking, giving likes, etc⋯) with social networks)). The pattern in the cases in which there were significant differences is quite similar, with younger subjects tending to use the smartphone behind the wheel more often than older ones, although with different nuances. Thus, in question a (Seeing who is calling you, without actually taking the call) the differences are found in the 18–24 vs. groups 44–54 and 55 or over, but not in intermediate ages, while in b (Answering or making a call not using the car’s hands-free device) the three younger age groups do this more frequently than the two older groups.

### 3.3. Predicting the Use of the Smartphone behind the Wheel Using Conspiracy Ideas

**Figure 1:**
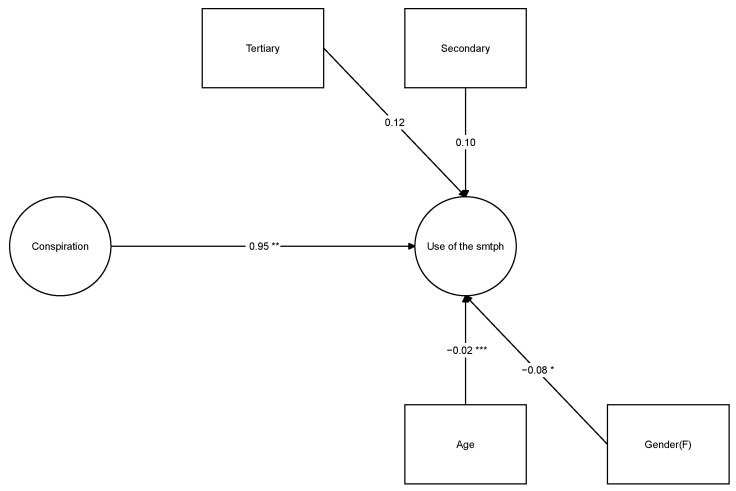
Coefficients of the regression model predicting smartphone use. * *p* < 0.05, ** *p* < 0.01, *** *p* < 0.001.

The ability to predict smartphone use behind the wheel using conspiracy ideas as the independent variable was tested using a structural equation model with the questions about conspiracy beliefs and mobile phone use as latent variables. In this model, gender, age (treated in this case as a numerical variable and not divided into categories as before), and the academic level of the participants as covariates were also introduced, since these variables have been shown to have an impact on the use of the smartphone behind the wheel. The two parts of this model, the measurement part and the structural part, can be consulted in Table 6 and Table 7. A graphic diagram of the structural part of model can be seen in Figure 1.

The model presented an adequate adjustment according to both the comparative fit index (CFI) and the Tucker–Lewis index (TLI): the former gave a value of 0.977 (above 0.9, which is considered to be the adequate value), and the latter gave a value of 0.981, which is also above the appropriate value of 0.9. Finally, the root mean square error of approximation was 0.038, with a significance value of 1 for the value of 0.05. The test of RMSEA is not significant, which means that we do not reject the null hypothesis that the RMSEA is less than or equal to 0.05. In this study, all factor loadings in the two latent variables reached statistical significance with *p*-values lower than 0.001. The indicator of composite reliability was computed from factorial loadings ω=0.67 for the scale about conspiracy ideas, and ω=0.82 for the scale about use of the smartphone at the wheel.

The structural part of the model shown in Table 7 has three significant coefficients: (a) the one corresponding to the latent variable underlying the conspiracy ideas, (b) the age of the subjects, and (c) gender. In the first case, a higher conspiracy score leads to greater use of the smartphone while driving, while increased age resulted in a negative coefficient. Women were associated with lower use of the smartphone at the wheel than men but the coefficient was not large.

## 4. Discussion

The results shown below suggest two main conclusions: (1) there are small percentages of people—between 3.9% and 11.5%—who agree with individual statements that we have called “conspiranoic beliefs” regarding smartphones at the wheel, but this percentage rises to 30% when we count people that least believe in at one of them, and (2) the latent or inferred variable underlying such conspiranoic beliefs can predict the reported used of the smartphone at the wheel. We will comment about these two conclusions below.

Banning the use of the smartphones at the wheel has not been completely successful in many cases, and the effects of the bans have often vanished as time passed [35,36,37] and may have even increased the risks associated with this behavior [30]. We have mentioned in the introduction that road safety measures and laws have often found opposition from groups of citizens disagreeing due to what Morelock et al. [9] called “myths”, because they felt that the government was not doing its work well [8], or because they were afraid that their individual liberties were at risk [7]. The percentage of people opposing the measures may vary in each case, but the results in Munnich Jr and Loveland [10] pointed that its size was often overemphasized by the media, and there were actually more citizens in support of the law than was represented in news coverage. However, in contrast with other road safety measures, there is not a organized civil movement fighting against the use of the smartphones at the wheel, but it seems that the percentage of people somewhat supporting it might be relatively large for one reason or another. In fact, taking into account the demographic variables used in this study, this support would be quite uniform between males and females, and among groups of age, although in depends on the academic level of the subjects, as those with low education expressed more agreement with many of the statements than the other groups.

The second of the results in this study suggests that conspiranoic beliefs about the smartphones at the wheel are able to predict their reported use, controlling by demographic covariates (gender, age, and level of the studies). Therefore, if confirmed by further research, conspiranoic beliefs should be added to list of the other factors that have already shown to be able to predict the use of smartphones at the wheel—normative beliefs of cellphone use, perceived risk of accident, perceived risk of fines, attitude toward using cellphones while driving, and perceived importance of calling, according to Yeo and Park [23]. It would be a matter of future research to see how these factors relate to each other, by perhaps causing, moderating, or mediating each other.

As noted by Van Prooijen and Jostmann [3], “conspiracy theories are consequential as they have a real impact on people’s health, relationships, and safety”. This statement assumes a causal relationship between maintaining conspiracy theories and the conduct of the subjects, which if applied to this specific case would lead to the assertion that conspiracy beliefs about smarphone use at the wheel can make the subjects who hold these beliefs behave more imprudently than those who do not, which results in crashes and/or injuries. There is plenty of evidence that links the distractions caused by the smartphones at the wheel with crashes, so it seems straightforward to connect the conspiracy beliefs to crashes throughout the use of the smartphone at the wheel as intermediate step. Future research should be able to prove such a relationship, but we should not jump to conclusions before such evidence is available.

With respect to applications of the results in this paper, as it is well known that conspiracy theories (myths in the words of Morelock et al. [9]) regarding road safety played a huge role in the past by delaying, hindering, or limiting the application of preventive measures that both industry and administrations claimed were beneficial to drivers [38], it is possible that the lessons learned about how to combat conspiracy theories in general could be applied to increase the acceptance of the bans of the use of the smartphone behind the wheel. Therefore, van Prooijen and van Vugt [39] mention three interventions or practical implications supported by the research on conspiracy theories: (1) providing rational arguments against specific theories reduces belief in them; (2) instilling feelings of security among the public as anxiety or lack of control increases belief in conspiracy theories [39,40,41]; (3) working on the social dimension of conspiracy theories, being careful for example that the groups more reluctant to adopt a recommendation do not perceive it as a menace specifically targeted to them.

Finally, we would like to mention as limitations of this study that the list of questions in what we have denominated conspiracy beliefs about the use of the smartphone at the wheel is probably still incomplete and could probably be expanded with in-depth interviews of selected subjects. Careful selection of the items used in this scale should improve its psychometric properties. We did not have the information about the specific device that drivers used in their cars, which could be a factor of importance in order to understand their answers. Furthermore, possible interactions between demographic variables and conspiracy beliefs could be relevant as some groups might experience higher effects on the use of the smartphone than others and consequently could be the focus of more specific interventions.

## Figures and Tables

**Table 1:** Demographics of the sample.

Variable	*N* = 1706
**Sex**	
Men	782 (46%)
Women	924 (54%)
**Age**	
18–24	159 (9.3%)
25–34	469 (27%)
35–44	542 (32%)
45–54	350 (21%)
Over 55	186 (11%)
**Level of education**	
University (Graduate, Doctor)	905 (53%)
High School	665 (39%)
Primary/No studies	136 (8.0%)
**Type of vehicle**	
Car	1601 (94%)
Motorbike	50 (2.9%)
Van	30 (1.8%)
Bus	19 (1.1%)
Truck	6 (0.4%)

Statistics presented: *n* (%).

**Table 2:** Conspiracy beliefs about using the smartphone while driving.

Indicate Whether You Agree with These Statements (I Agree/I Do Not Agree):
1. Talking on the smartphone does not affect driving
2. I do not think that looking at the smartphone makes you drive worse
3. You do not lose sight of the road when using your smartphone behind the wheel
4. Fines for using the smartphone are only for tax collecting purposes
5. You must not turn on your smartphone when you drive because, if you do so, they can
pinpoint your location
6. The statistics of accidents caused by smartphone use are all made up
7. By not being able to use the smartphone, people become more bored when driving
which leads to more accidents
8. If you go alone on the road and it is straight there is no danger in using
your smartphone

**Table 3:** Questions about frequency of use of the smartphone behind the wheel.

Question
**In the last twelve months, indicate how often you have used your smartphone to perform these actions WHILE DRIVING (1 = Never, 2 = Almost never, 3 = Sometimes, 4 = Almost always, 5 = Always)**
a. Seeing who is calling you, without actually taking the call.
b. Answering or making a call not using the car’s hands-free device.
c. Answering or making a call by putting on the smartphone speaker.
d. Answer or make a call using the car’s hands-free device.
e. Reading a WhatsApp message or something similar or an email.
f. Sending a WhatsApp message or something similar or an email.
g. Interacting (checking, giving likes, etc…) with social networks.
h. Using or handling the smartphone’s GPS navigator.
i. Taking selfies or making recordings with the smartphone.
j. Using for entertainment purposes: selecting music, playing games, reading news,
searching the internet, shopping.

**Table 4:** Frequencies and percentages of subjects who answered that they agreed with the statements listed in Table 1. The *p*-values are the result of chi-square or Fisher’s exact tests depending on the number of cells.

Question	*N* (%)	Gender *N* (%)			Academic Level *N* (%)				Age *N* (%)					
	**Total**	**Men**	**Female**	***p***	**Tertiary**	**Secondary**	**Primary/No Stud.**	***p***	**18–24**	**25–34**	**35–44**	**44–54**	**55 or More**	***p***
1	129 (7.6)	65 (8.3)	64 (6.9)	0.324	57 (6.3)	57 (8.6)	15 (11)	0.068	13 (8.2)	43 (9.2)	27 (5)	29 (8.3)	17 (9.1)	0.095
2	111 (6.5)	52 (6.6)	59 (6.4)	0.903	38 (4.2) a	53 (8) b	20 (14.7) b	<0.001	13 (8.2) ab	27 (5.8) b	29 (5.4) b	16 (4.6) b	26 (14) a	<0.001
3	83 (4.9)	37 (4.7)	46 (5)	0.902	35 (3.9) a	35 (5.3) ab	13 (9.6) b	0.013	8 (5)	21 (4.5)	28 (5.2)	13 (3.7)	13 (7)	0.544
4	123 (7.2)	78 (10)	45 (4.9)	<0.001	53 (5.9)	56 (8.4)	14 (10.3)	0.053	7 (4.4) a	35 (7.5) a	29 (5.4) a	34 (9.7) a	18 (9.7) a	0.046
5	196 (11.5)	95 (12.1)	101 (10.9)	0.478	85 (9.4) a	86 (12.9) ab	25 (18.4) b	0.003	17 (10.7)	48 (10.2)	59 (10.9)	43 (12.3)	29 (15.6)	0.365
6	79 (4.6)	48 (6.1)	31 (3.4)	0.009	29 (3.2) ac	40 (6) bc	10 (7.4) c	0.009	4 (2.5)	18 (3.8)	27 (5)	19 (5.4)	11 (5.9)	0.459
7	92 (5.4)	48 (6.1)	44 (4.8)	0.252	31 (3.4) a	43 (6.5) b	18 (13.2) c	<0.001	7 (4.4)	25 (5.3)	29 (5.4)	21 (6)	10 (5.4)	0.967
8	67 (3.9)	33 (4.2)	34 (3.7)	0.655	30 (3.3)	30 (4.5)	7 (5.1)	0.361	9 (5.7)	19 (4.1)	22 (4.1)	9 (2.6)	8 (4.3)	0.544
One ^a^	510 (30)	256 (33)	254 (27)	0.02	234 (26) a	220 (33) b	56 (41) bc	<0.001	42 (9)	139 (26)	146 (42)	112 (60)	71 (9)	0.709
Total	1706 (100)	782 (46)	924 (54)		905 (53)	665 (39)	136 (8)		159 (9)	469 (27)	542 (32)	350 (21)	186 (11)	

abc: Proportions in a row without a common superscript letter differ *p* < 0.05 according to Holm’s adjusted post-hoc pairwise proportion tests. 1. Talking on the smartphone does not affect driving; 2. I do not think that looking at the smartphone makes you drive worse; 3. You do not lose sight of the road when using your smartphone behind the wheel; 4. Fines for using the smartphone are only for tax collecting purposes; 5. You must not turn on your smartphone when you drive because, if you do so, they can pinpoint your location; 6. The statistics of accidents caused by smartphone use are all made up; 7. By not being able to use the smartphone, people become more bored when driving which leads to more accidents; 8. If you go alone on the road and it is straight there is no danger in using your smartphone; ^a^ Respondents that agreed with at least one of the statements 1–8.

**Table 5:** Means and standard deviations of the responses to the statements listed in Table 2. *p*-values are the result of *t*-tests or analysis of variance tests depending on the number of categories of the variable.

Question	All	Gender				Academic Level					Age						
	**Total**	**Men**	**Female**	***p***	**d**	**Tertiary**	**Secondary**	**Pr./No Stud.**	***p***	**Eta**	**18–24**	**25–34**	**35–44**	**44–54**	**55 or More**	***p***	**Eta**
1	2.1 (1.08)	2.0 (1.1)	2.1 (1.1)	0.23	0.06	2.1 (1.1) a	2.0 (1.1) a	1.8 (1.0) b	<0.01	0.03	2.4 (1.1) a	2.2 (1.1) ab	2.1 (1.1) bc	2.0 (1.1) c	1.6 (0.9)d	<0.01	0.11
2	1.4 (0.74)	1.4 (0.7)	1.4 (0.7)	0.69	0.02	1.4 (0.7)	1.4 (0.8)	1.4 (0.7)	0.43	0.00	1.5 (0.9) a	1.5 (0.8) a	1.5 (0.8) a	1.3 (0.6) b	1.3 (0.6) b	<0.01	0.04
3	1.8 (0.99)	1.7 (1.0)	1.8 (1.0)	<0.01	0.14	1.8 (1.0)	1.8 (1.0)	1.6 (1.0)	0.095	0.01	2.2 (1.2) a	1.9 (1.0) a	1.7 (1.0) b	1.6 (0.9) b	1.3 (0.7) c	<0.01	0.14
4	2.5 (1.45)	2.7 (1.5)	2.5 (1.4)	<0.01	0.15	2.6 (1.5)	2.6 (1.4)	2.3 (1.5)	0.25	0.01	2.6 (1.4) ab	2.7 (1.4) a	2.6 (1.5) a	2.5 (1.5) a	2.2 (1.4) b	<0.01	0.03
5	1.6 (0.9)	1.6 (0.9)	1.7 (0.9)	0.54	0.03	1.7 (0.9) a	1.6 (0.9) ab	1.4 (0.9) b	<0.01	0.03	1.8 (1.0) ab	1.9 (1.0) a	1.6 (0.9) bc	1.5 (0.8) c	1.2 (0.6)d	<0.01	0.17
6	1.5 (0.81)	1.4 (0.8)	1.5 (0.8)	0.11	0.08	1.5 (0.8)	1.5 (0.8)	1.4 (0.8)	0.081	0.01	1.6 (0.9)	1.6 (0.9) ab	1.7 (0.9) a	1.5 (0.8) bc	1.3 (0.7) c	<0.01	0.17
7	1.1 (0.49)	1.2 (0.5)	1.1 (0.4)	0.051	0.10	1.1 (0.5)	1.1 (0.5)	1.1 (0.4)	0.83	0.00	1.2 (0.6)	1.1 (0.4)	1.1 (0.5)	1.1 (0.5)	1.1 (0.4)	0.8	0.00
8	1.6 (0.9)	1.7 (0.9)	1.6 (0.9)	0.29	0.05	1.7 (0.9) a	1.6 (0.9) b	1.5 (0.9) ab	0.02	0.02	1.7 (1.0) ab	1.8 (1.0) a	1.7 (0.9) ab	1.5 (0.8) b	1.3 (0.6) c	<0.01	0.11
9	1.1 (0.45)	1.1 (0.4)	1.1 (0.5)	0.89	0.01	1.1 (0.4) a	1.2 (0.5) b	1.1 (0.6) ab	0.022	0.02	1.2 (0.6) a	1.1 (0.4) ab	1.1 (0.4) ab	1.1 (0.4) b	1.1 (0.4) ab	0.02	0.02
10	1.3 (0.67)	1.3 (0.7)	1.3 (0.7)	0.76	0.01	1.2 (0.6)	1.3 (0.7)	1.3 (0.7)	0.62	0.00	1.4 (0.8) a	1.3 (0.8) a	1.2 (0.7) ab	1.2 (0.6) bc	1.1 (0.4) c	<0.01	0.07

abc: Means in a row without a common superscript letter differ *p* < 0.05 according to Games-Howell post hoc tests using the Tukey’s correction.1. Seeing who is calling you, without actually taking the call; 2. Answering or making a call not using the car’s hands-free device; 3. Answering or making a call by putting on the smartphone speaker; 4. Answer or make a call using the car’s hands-free device; 5. Reading a WhatsApp message or something similar or an email; 6. Sending a WhatsApp message or something similar or an email; 7. Interacting (checking, giving likes, etc…) with social networks; 8. Using or handling the smartphone’s GPS navigator; 9. Taking selfies or making recordings with the smartphone; 10. Using for entertainment purposes: selecting music, playing games, reading news, searching the internet, shopping.

**Table 6:** Loadings of items in the two scales. Fit indexes are: SRMR = 0.054, CFI = 0.975, TLI = 0.974, RMSEA = 0.038 *p* (≤0.05) = 1.

Question	coef	s.e	*t*	*p*	Stand. Load
**Conspiranoic ideas**					
1	1.000	0.000			0.555
2	1.013	0.209	4.855	<0.001	0.606
3	0.714	0.136	5.265	<0.001	0.487
4	0.665	0.151	4.406	<0.001	0.378
5	0.487	0.134	3.634	<0.001	0.224
6	0.605	0.131	4.611	<0.001	0.423
7	0.509	0.117	4.347	<0.001	0.332
8	0.739	0.137	5.405	<0.001	0.558
**Use of the smartphone**					
1	1.000	0.000			0.692
2	1.043	0.035	30.156	<0.001	0.720
3	1.047	0.033	32.137	<0.001	0.723
4	0.612	0.037	16.431	<0.001	0.430
5	1.307	0.033	39.511	<0.001	0.890
6	1.314	0.033	40.379	<0.001	0.894
7	1.134	0.040	28.655	<0.001	0.779
8	0.890	0.033	27.025	<0.001	0.619
9	1.012	0.047	21.557	<0.001	0.700
10	0.906	0.042	21.649	<0.001	0.630

**Table 7:** Structural model for the use of the mobile phone.

Predictor	coef	s.e	*t*	*p*	β
Conspiration beliefs	0.955	0.308	3.099	0.002	0.197
Age	−0.019	0.002	−10.325	<0.001	−0.308
Gender(F)	−0.084	0.039	−2.140	0.032	−0.059
Tertiary	0.120	0.076	1.579	0.114	0.085
Secondary	0.104	0.077	1.346	0.178	0.072

## Data Availability

The data presented in this study are available on request from the corresponding author.
